# A multimodal intervention program to control a long-term *Acinetobacter baumannii* endemic in a tertiary care hospital

**DOI:** 10.1186/s13756-019-0658-4

**Published:** 2019-12-04

**Authors:** R. Valencia-Martín, V. Gonzalez-Galan, R. Alvarez-Marín, A. M. Cazalla-Foncueva, T. Aldabó, M. V. Gil-Navarro, I. Alonso-Araujo, C. Martin, R. Gordon, E. J. García-Nuñez, R. Perez, G. Peñalva, J. Aznar, M. Conde, J. M. Cisneros, In representation of A. baumannii eradication program

**Affiliations:** 1 Departments of Infectious Diseases, Microbiology, and Preventive Medicine, University Hospital Virgen del Rocío–Institute of Biomedicine of Seville, Avenida Manuel Siurot s/n, 41013 Seville, Spain; 2 Department of Intensive Care, University Hospital Virgen del Rocío, Seville, Spain; 3 Department of Pharmacy, University Hospital Virgen del Rocío, Seville, Spain; 4 Cleaning Service, University Hospital Virgen del Rocío, Seville, Spain; 5 University Hospital Virgen del Rocío, Seville, Spain

**Keywords:** *Acinetobacter baumannii*, Infection control, Outbreak, Endemic

## Abstract

**Background:**

*Acinetobacter baumannii* causes frequently nosocomial infections worldwide. Its ability to survive on dry surfaces facilitates its spread and the persistence of endemic situations, especially in the intensive care units (ICUs).

The objective of this paper is to describe a multicomponent intervention program designed to control a hyperendemic persistence of multidrug-resistant *A. baumannii* (MDR-Ab) and to characterize its impact.

**Methods:**

Design: Quasi-experimental intervention study based on open cohorts.

Setting: Public tertiary referral centre. Period: January 2009–August 2017.

Intervention: multifaceted program based on environmental decontamination, hand hygiene, antimicrobial stewardship, contact precautions, active surveillance, weekly reports and regular meetings.

Analysis: joinpoint regression and interrupted time-series analysis.

**Results:**

The intervention was successfully implemented. Through the study period, the compliance with contact precautions changed from 0 to 100% and with hand hygiene, from 41.8 to 82.3%. Between 2012 and 2016, the antibiotic consumption decreased from 165.35 in to 150.44 daily-defined doses/1000 patients-days in the ICU. The incidence density of MDR-Ab in the ICU was 10.9 cases/1000 patients-days at the beginning of the intervention. After this moment, the evolution of the incidence density of MDR-Ab was: between months 0 and 6°, it remained stable; between months 7° and 10°: there was an intense decrease, with an average monthly percentage change (AMPC) = − 30.05%; from 11° month until the end, the decrease was lighter but continuous (AMPC:-2.77%), achieving an incidence density of 0 cases/1000 patients-days on the 18° month, without any new case for 12 months. From the 30° month until the end of the study period, several little outbreaks of MDR-Ab were detected, all of them rapidly controlled. The strains of MDR-Ab isolated during these outbreaks were not clonally related with the previously endemic one, which supports its eradication from the environmental reservoirs.

**Conclusion:**

The multicomponent intervention performed by a multidisciplinary team was effective to eradicate the endemic MDR-Ab.

## Background

*Acinetobacter baumannii* is a frequent etiology of nosocomial infections worldwide, posing a major challenge due to its great ability to develop resistance against antibiotics [1, 2]. In recent years, most reported clinical isolates are multidrug-resistant, and several outbreaks caused by extremely-drug resistant (XDR) or even pan-drug resistant (PDR) have been described [3–5]. Hospital outbreaks and endemic situations are expedited by the capacity of *A. baumannii* to survive on all kind of surfaces [6]. However, although some outbreaks could be controlled by eliminating an environmental reservoir [7], in many centres all over the world affected by endemic situations with occasional superimposed outbreaks [8–11], endemicity tipically persists after controlling the outbreaks flaring up. This problem affects especially to Intensive Care Units (ICUs) [2, 12].

Our hospital experienced a two decades-long hyperendemic situation with *A. baumannii* and even an outbreak of PDR-*A. baumannii* in 2002 [5]. Since 2008, most clinical isolates were carbapenem-resistant, all of them susceptible to colistin. Despite several interventions, including hand hygiene, contact precautions and an antimicrobial stewardship program, the incidence of multidrug resistant *A. baumannii* (MDR-Ab) did not decrease. In late 2012, a second outbreak of XDR-*A. baumannii*-resistant to colistin was declared in the adults ICU.

After the epidemiological investigation and environmental and surveillance cultures, a molecular characterization of the XDR isolates by pulsed-field gel electrophoresis (PFGE) [13] confirmed that they were closely related, according to the Tenover criteria [14]. With the purpose of facing this situation, we designed an alternative interventional program to control the outbreak and the endemic situation. On this paper, we report the multicomponent program developed and its impact on the incidence of MDR-Ab.

## Methods

### Setting

University Hospital Virgen del Rocío (Seville, Spain) is a 1367 bed-centre with two adult ICUs, which have six multibed open wards for medical and surgical patients (50 beds), and two wards for trauma and neurosciences areas divided into rooms for one patient each (18 beds). This is a reference centre for severe trauma, neurosurgery, burn patients and solid organ and bone marrow transplantations. During the studied period, the hospital and ICUs had, respectively, an average of 52,593 and 3379 inpatient episodes per year.

### Study design

This is a quasi-experimental intervention study, based on open cohorts of all the patients admitted to the hospital from the 1st January 2009 to the 31^st^August 2017, having a pre-intervention period of 46 months and a post-intervention period of 58 months.

### Infection control team

A multidisciplinary team, composed of an infection control and hospital hygiene specialist, a microbiologist, a pharmaceutical specialist, an infectious diseases physician and three nurses with expertise in infection control, designed and led the intervention program.

### Intervention program

A multifaceted program was implemented. All the measures agreed the current recommendations of the Centers for Disease Control and Prevention (CDC) [15].

#### Environmental decontamination

An exhaustive environmental cleaning policy was instituted. The disinfectant used were a hypochlorite-based disinfectants; for the handling of not-disposable medical products in which this disinfectant was not appropriate, a wipe disinfectant containing benzalkonium chloride and propane-1,2-diol was used. Medical products were exclusive of each colonized of infected patient. Disposable medical products were discarded once the patient did not need them any more following the CDC recommendations [16], and not-disposable products were cleaned following the procedures previously described.

To verify the environmental decontamination procedure, two specific checklists were created: one for the general environmental cleaning and the other for medical equipment disinfection. These checklists had to be filled by the cleaning staff every time that any cleaning procedure was performed, and at least twice each day; the percentage of compliance was monitored weekly. The infection control team met several times with the cleaning staff for educational purposes and feedback of the results.

Every ICU ward was sequentially closed for a terminal cleaning at the beginning of the program, and periodically at least thrice a year. Afterwards, every bedside of the ICU and the rooms in other wards from which a colonized patient was discharged underwent terminal cleaning as well.

#### Hand hygiene instruction and surveillance

Hand hygiene education courses were provided to the ICU staff the first weeks after the XDR-Ab outbreak, and periodically afterwards, emphasizing on training on newly incorporated staff. The hand hygiene training was based on the performance of periodic workshops in which the five moments for hand hygiene were remembered and the technique was trained using an UV glow box. During the first year, structured observation of hand hygiene compliance during the daily care activity was performed weekly by the nurses of the infection control team in the ICU, following the recommendations and observation tool of the WHO [17]. Afterwards, this observation was performed periodically (at least once each month). The percentage of compliance was included in the weekly reports.

#### Antimicrobial stewardship program

Until the outbreak of XDR-Ab was controlled, colistin was restricted, needing pre-authorization and being revised the indication and duration of every colistin course. Afterwards, the educational stewardship program previously set in the hospital [18] was enhanced in the ICUs, adding a weekly feedback of the antibiotic consumption data to the staff.

#### Isolation and contact precautions

Contact precautions, following the CDC recommendations [16], were already mandatory for patients carrying MDR-Ab. In addition, we implemented the surveillance of its compliance and displayed information posters in every affected unit.

Contact precautions were maintained through the whole admission period for patients with MDR-Ab.

#### Active surveillance for MDR-Ab colonization

All patients admitted to adult ICUs were screened weekly. Surveillance cultures were obtained by rectal and pharyngeal swabs.

#### Weekly report

A weekly report was made with the evolution of the selected indicators. It includes the level of compliance of the measures and the results achieved. Definitions and indicators are described below.

#### Regular meetings with the staff of the affected areas

The infection control team met weekly with the staff of the ICUs (physicians, nurses, assistant nurses, hospital attendants, housekeepers) to discuss the results of the weekly report, and to take additional measures when necessary. Additionally, the medical and nurse directors were informed by weekly email for the whole period.

### Definitions and indicators

*A. baumannii* was defined as MDR or XDR following the criteria of Magiorakos et al. [19], which consider that *A. baumannii* is MDR when non-susceptible to ≥1 agent in ≥3 antimicrobial categories (including aminoglycosides, carbapenems, fluorquinolones, antipseudomonal penicillins+β-lactamases inhibitors, extended spectrum cephalosporins, trimethoprim-sulphamethoxazole, ampicillin-sulbactam, polymixins and tetracyclines) and XDR when non-susceptible to ≥1 agent in all but ≤2 of the same categories. If the susceptibility to one antimicrobial was not tested, it was classified as “resistant” (see below). The main outcome was the incidence density of MDR-Ab in clinical samples, defined as the number of patients newly infected or colonized with MDR-Ab per 1000 patient-days. Surveillance samples were excluded because of their absence in the pre-intervention period. An isolate of MDR-Ab was considered as a new episode after 365 days of the last positive culture in the same patient or when the initial infection was cured and MDR-Ab was not isolated in three rectal swabs performed in three consecutive weeks. Hand hygiene compliance was expressed as the percentage of correct actions over all the observed ones [20]. The appropriateness of the isolation and contact precautions was evaluated with a form designed for this purpose. The global compliance was the percentage of patients with a correct response to all the items over the total of patients under contact precautions observed. Environmental and medical equipment cleaning compliance was evaluated by checklists designed for this purpose, completed by the responsible staff (housekeepers and assistant nurses, respectively). They were stated as the percentage of correct actions of each item over the total. Antimicrobial consumption was measured in Daily Defined Doses (DDD) per 1000 patients-days. Hand hygiene, contact precautions and cleaning compliance were surveyed just in the ICUs; all the others in the whole hospital, analysing separately the ICUs.

### Microbiological procedures

Active surveillance for MDR-Ab colonization was performed in all patients admitted to the ICU with at least prior 48 h hospitalization [21]. The culture media used was *Brilliance*™ CRE Agar, a chromogenic screening plate for the detection of carbapenem-resistant strains with high sensitivity and specificity and whose results are available in just 18–24 h. Each isolate was identified by the Matrix-assisted laser desorption/ionization time of flight mass spectrometry (MALDI-TOF MS- Brucker®). Susceptibility testing to colistin and meropenem of the isolates was studied by E-test© (AB Biodisk, Sweden) according to the manufacturer’s recommendations. Susceptibility to other antibiotics was performed by using commercial microdilution methods (PMicroScan combo NC58. BeckamCoulter. USA). Amikacin, ampicillin/sulbactam, imipenem, minocycline and tigecycline were tested in all the isolates. Due to the generalized resistance to some groups of antibiotics (fluorquinolones, antipseudomonal penicillins+β-lactamases inhibitors, extended spectrum cephalosporins, trimethoprim-sulphamethoxazole and tetracycline) among the isolates of *A. baumannii* from our hospital in previous years, the susceptibility to them was tested just in a variable proportion of isolates. The breakpoints used were those recommended by EUCAST in each time frame [22].

Molecular typing using PFEG was performed for the initial outbreak investigation, as mentioned above, and to the new isolates of the outbreaks that occurred after the eradication of the endemic. Plug preparation, lysis, cell washing, restriction digestion (60 U of *Apa*I), and electrophoresis were performed as previously described [13]. PFGE was performed by using a clamped homogeneous electric field electrophoresis (CHEF) DRIII apparatus (Bio-Rad Laboratories, Hercules, CA). The conditions employed were as follows: temperature of 14 °C, voltage of 6 V/cm, run time of 19 h, and switch time of 5 to 20 s. The images obtained were processed with Bio- Rad Molecular Imager® GelDoc™ XR+ with Lab™ Software. PFGE clustering was determined by using the unweighted-pair group method with arithmetic averages (UPGMA) and by using Dice’s coefficient. The tolerance was set at 1%. All calculations were performed by using InfoQuest software (Applied Maths, Saint-Martens-Latem, Belgium). The results of the PFGE typing were compared according to the Tenover criteria [14].

### Statistical analysis

To estimate changes in the observed trends we used a joinpoint regression analysis [23], with previous evaluation of homocedasticity and existence of autocorrelation for each variable using SPSS Version 19.0 (Armonk, NY: IBM Corp.). These models give a double result: they identify the time point in which the trend changes and they also estimate the observed trend in each time interval. A maximum of three turning points were searched for each regression analysis. Statistical significance was set in an alpha error of 0.05. The software used was Joinpoint Regression Program, Version 4.5.0.1 - June 2017 (Statistical Methodology and Applications Branch, Surveillance Research Program, National Cancer Institute, Bethesda, MD, USA). In addition, we performed an interrupted time series (ITS) analysis with R version 3.4.3.

## Results

### Rates of MDR-Ab colonization and infection

**Fig. 1:**
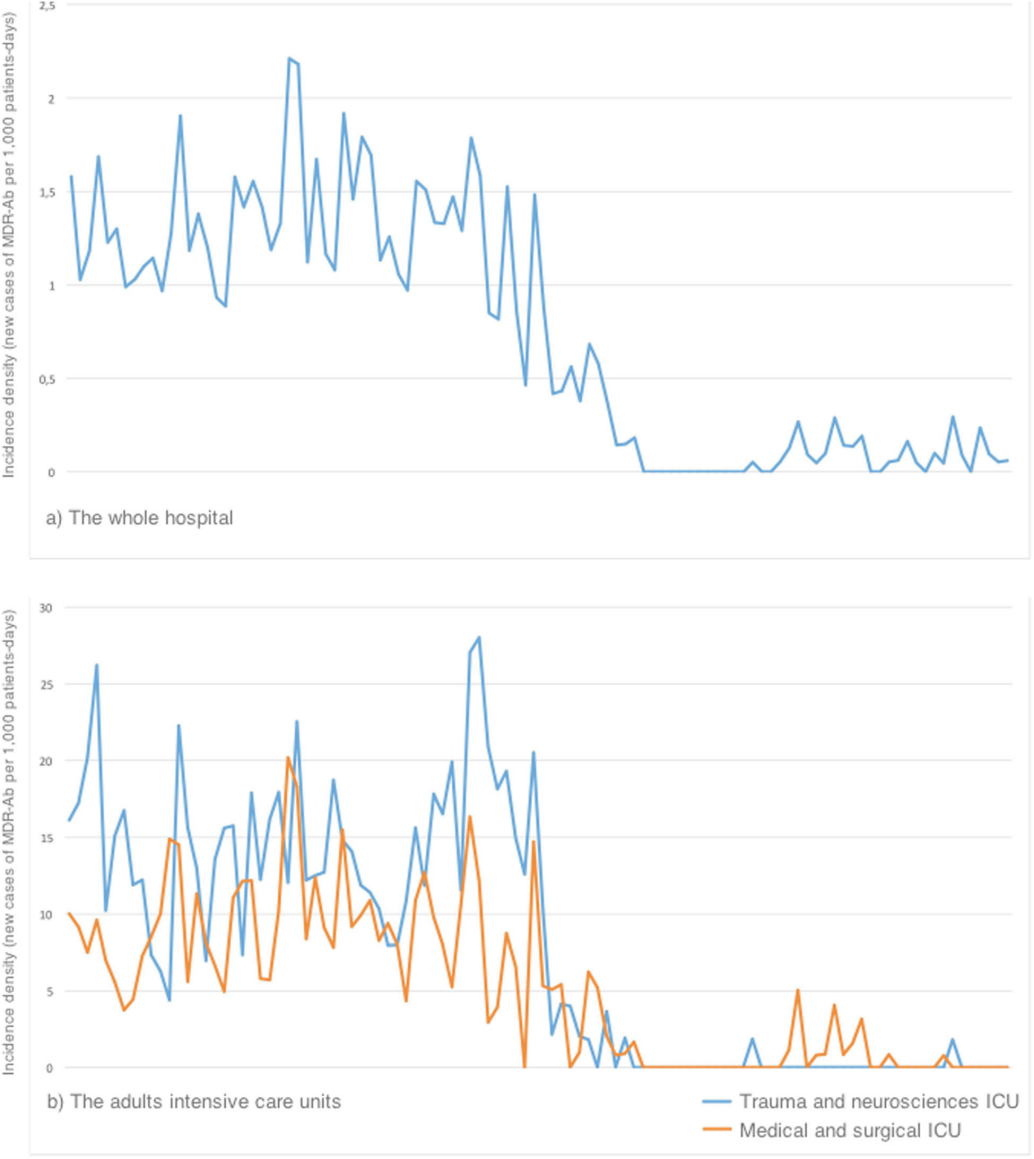
Evolution of the incidence density rates of multidrug resistant *A. baumannii*: **a**) the whole hospital; **b**) the adults intensive care units

**Fig. 2:**
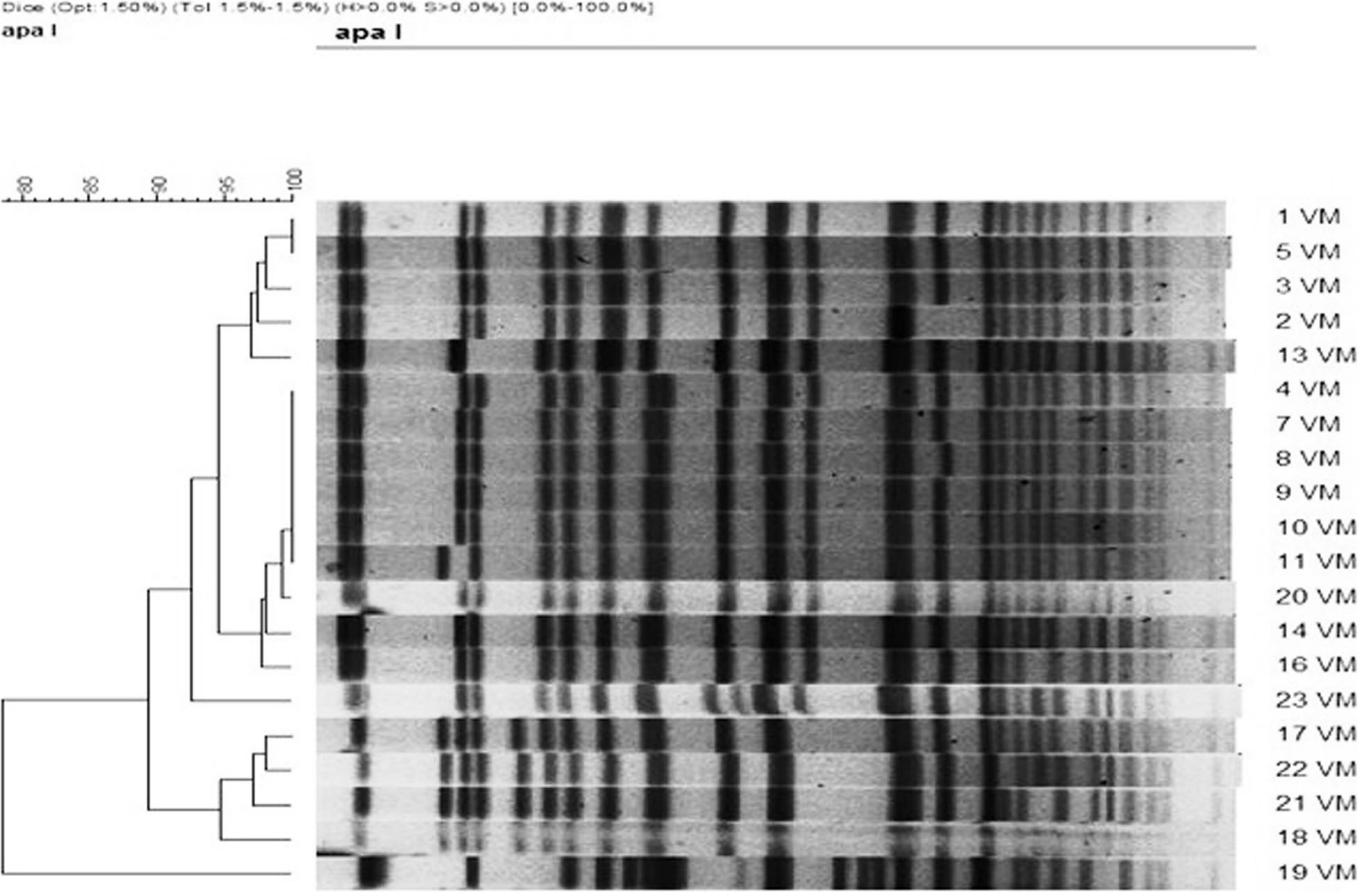
PFGE profiles of *Apa*I-digested genomic DNA from clinical and environmental strains of *A. baumannii*

The outbreak of XDR-Ab affected five patients and was controlled in three weeks. The incidence density of MDR-Ab was 10.9 cases/1000 patients-days at the beginning of the enhanced infection control program. The monthly tracking of this incidence is represented in Fig. 1. Sixty weeks after the multifaceted program started, the incidence achieved 0 cases/patients-days in the ICUs. No new cases were described for 34 weeks since week 94. Later, there have been several outbreaks after the eradication of the endemic situation, caused by patients previously colonized at the moment of the admission (Fig. 1). On Fig. 2, PFGE shows the restriction profiles of genomes extracted from isolates causing outbreaks occurred in 2015 and compares to those caused in 2009. The differences on bands that are observed among the lanes indicate that the isolates causing these outbreaks were distinct from those of the preceding years at level of their genomes, applying Tenover criteria [14].

The incidence density of MDR-Ab bacteraemia at the beginning of the program was 0.026 cases/1000 patients-days, with a crude mortality of 50%. Three trimesters later, the incidence achieved 0.000 cases/1000 patients-days and no new cases of MDR-Ab bacteraemia have been observed.

**Fig. 3:**
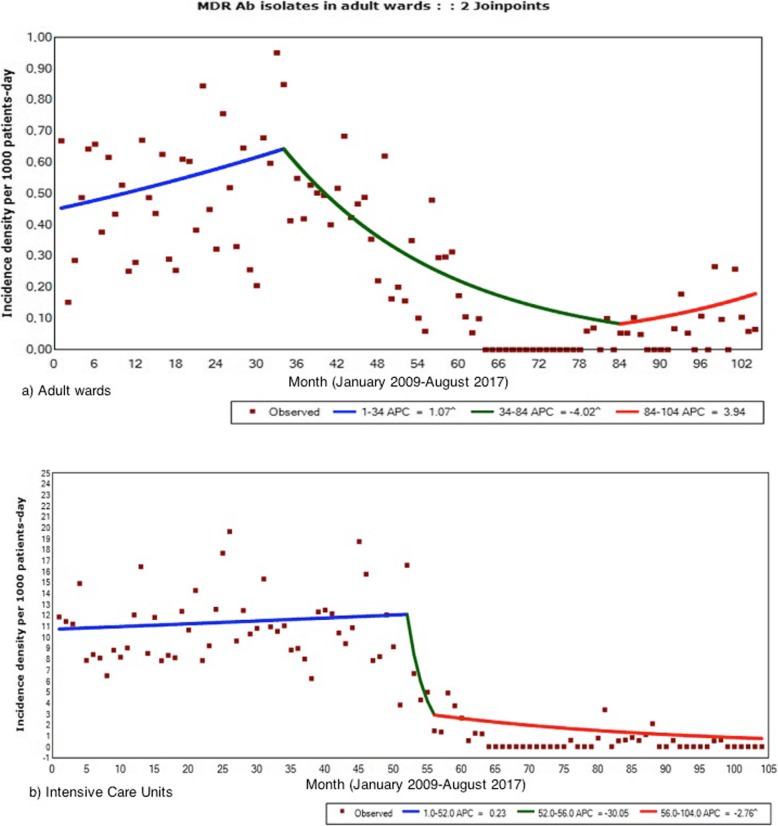
Joinpoint regression analysis of the incidence density of multidrug resistant *A. baumannii*: **a**) the adult wards; **b**) the adult intensive care units

The joinpoint regression analysis of the MDR-Ab rates is given in Table 1. This analysis selected the beginning of the multifaceted intervention as the point of trend change, either in the wards or the ICU (Fig. 3). In the ICU there was an upward baseline trend (AMPC: 0.23%); 6 months after the intervention (month 52) we observed a trend change followed by an intense decrease during four months (AMPC: − 30.05%), continuing with a significant tendency towards zero (AMPC: − 2.77%) until the end of the period. In the rest of adult wards the AMPC underwent a change prior to the implementation of the program in month 34 (1.07%), followed by a prolonged decline (AMPC: − 4.02%) maintained for 18 months, moving to a final trend not statistically significant (AMPC: 3,94%).

**Table 1:** Changes in rates of multidrug resistant *Acinetobacter baumannii* infection and colonization: results from the joinpoint regression analysis

Settings	Incidence Rates MDR-Ab				
	Pre-intervention trend	Point of change 1	Post 1 trend	Point of change 2	Post 2 trend
	AMPC [CI95%] (*p*-value)	Month [CI95%] *p* < 0.05	AMPC [CI95%] (*p*-value)	Month [CI95%] *p* < 0.05	AMPC [CI95%] (*p*-value)
Adults hospital (ICU + wards)	0.21%[−0.40;0.82] (*p* = 0.50)	49 [41;54] (Jan/2013)	−12.14% [−17.48;-6.46] (*p* = 0.001]	67 [53;84] (Jul/2014)	0.46 [−4.05;5.18] (*p* = 0.84)
ICU	0.23%[−0.25;0.71] (*p* = 0.34)	52 [50;54] (Apr/2013)	−30.05%[−55.10;8.98] (*p* = 0.11)	56 [54;67] (Aug/2013)	−2,77% [−4.91;-0.57] (*p* = 0.01)
Adults wards	1.07% [0.01;2.13] (*p* = 0.05)	34 [30;50] (Oct/2011)	−4.02% [−5.37;-2.65] (*p* = 0.001)	84 [61;95] (Dec/2015)	3.94 [−2.40;10.70] (0.23)

*AMPC* Average monthly percentage change, *CI* confidence interval, *MDR-Ab* multidrug resistant *Acinetobacter baumannii*, *ICU* intensive care unit

**Fig. 4:**
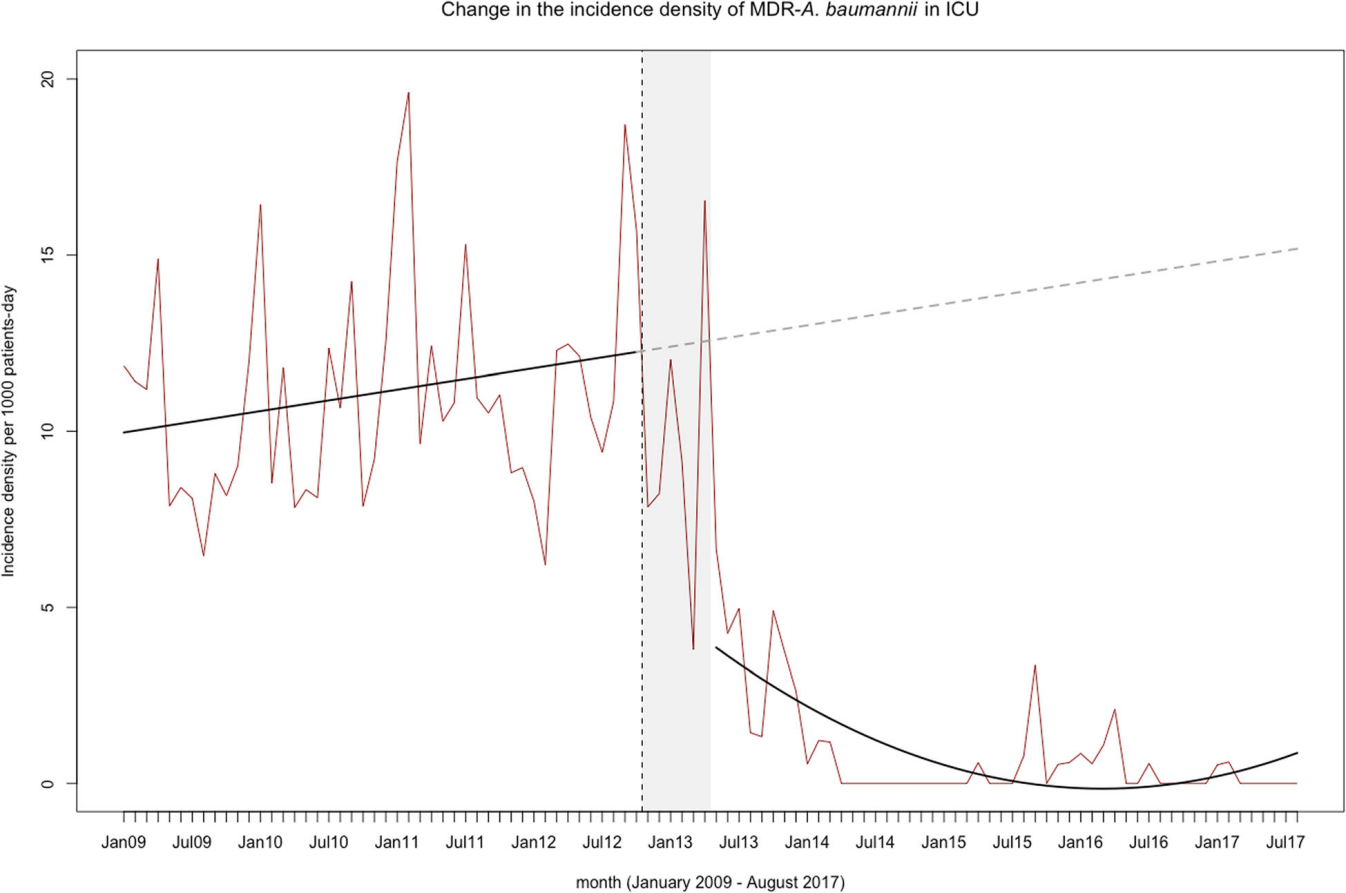
Interrupted time series analysis with transition 6-month period of the incidence density of multidrug resistant *A. baumannii* in the intensive care units

For the interrupted time series analysis, we considered a 6-month lag to reach endemic control (time necessary to decrease the incidence density rates of 5‰). In the ICU we observed an increasing baseline trend followed, after the phase-in period, by a decreasing non-linear trend throughout the rest of the period (Fig. 4).

### Environmental decontamination

The first weeks, the overall environmental cleaning compliance was 83%. After 14 weeks, a 100% of compliance in all the items of the checklists was achieved.

### Contact precautions and hand hygiene

The first evaluation revealed that the adherence to all the indicated measures of contact precautions was correct in 0% of the cases. Although all the colonized/infected patients were visibly identified, the individual protection equipment was correctly located just in 5.6% of them and only 22.2% received the appropriate hygiene, while the visits policy was systematically incorrect. Two years later, the compliance with the contact precautions was 100% in all the evaluated items.

The compliance with the hand hygiene was 41.8% in the first surveillance campaign. Until October 2014, after 702 observations, the average rate of hand hygiene adherence had been 82.3%. Nonetheless, adherence rates have been varying throughout the study period and are closely related to changes in the staff.

### Surveillance cultures

During the study period, between 80 and 100 weekly samples were processed and 169 patients were colonized by MDR-Ab. The incidence of newly colonized patients decreased along with the isolation of MDR-Ab from clinical samples, as shown in Fig. 1. From 31/12/2016 to 31/08/2017, no cases of nosocomial acquisition of MDR-Ab were detected by surveillance cultures.

### Antibiotic consumption

The antibiotics consumption decreased during the study period, as we previously reported [24]. The global antibiotic consumption in the ICU decreased from 165.35 DDD/1000 to 150.44 DDD/1000 patients-days between 2012 and 2016, while the consumption of colistin reduced from 25.96 to 6.65 DDD/patients-days during the same period. The consumption of carbepenems also decreased notably (from 31.47 to 15.9 DDD/1000 patients-days), with moderate increase of families with less spectrum of activity, as penicillins, penicillins/β-lactamase inhibitors and cephalosporins. (Evolution of the consumption of diverse antimicrobials in the ICUs from the beginning of the whole intervention to the end of the study is shown in Additional file 1).

## Discussion

A two-decade intense endemic setting of MDR-Ab can be eradicated. To achieve this success, we needed a long-term multifaceted program, led by a multidisciplinary infection-control team, and involving the hospital management and all the concerned staff.

The efficacy of this kind of approaches is widely supported by previous reports [25–32], with small differences among them in the measures registered, adapted to the local particularities. Many authors have reported a complete control of MDR-Ab outbreaks in hospitals where this rod was not previously present [28, 30–32], and many others described the efficacy of infection control programs to significantly reduce the incidence of MDR-Ab in centres with long-term endemic situations [25, 26, 29]. Moreover, a PDR-Ab outbreak was successfully controlled in 2002–03 by a multicomponent approach in our hospital, but it was unable to reach a complete eradication of MDR-Ab [5].

Furthermore the multifaceted approach, currently mandatory, there were several aspects that probably enabled these results.

First of all, there is the “human factor”, that must be considered as the essential element on the multifaceted program: a strong formation on antimicrobial stewardship and infection disease management is required, with the necessary knowledge about how to lead the intervention; the unconditional institutional support from the Medical Director and Director of Nurses of both the hospital and the affected units; and also to keep the meetings with the staff, either for education or feedback.

Secondly, the relevance of keeping the actions along the time. Despite all the efforts, we needed 6 months to control the endemic situation, 60 weeks to appreciate an improvement on the incidence of MDR-Ab and 94 weeks to maintain these results. The persistence of reservoirs (colonized patients, environmental sources) and the lack of compliance of hand hygiene or contact precautions can explain this discouraging phenomenon, described in most successfully controlled MDR-Ab endemic situations [25]. Thus, infection control teams that want to face these situations may be aware: immediate effects are not expectable, and achieving successful significant results with multifaceted programs needs a long implementation period. A relevant impact will probably appear after several months of keeping to the measures and with periodical evaluation of possible errors of compliance. In addition, the occurrence of outbreaks of MDR-Ab after the eradication of the endemic situation, caused either by community patients previously colonized or patients transferred from other hospitals is almost warranted; which is another reason to maintain the continuance of the program.

The bundle applied was big and complex, but many of its measures were on going previously. Why were not they working until the whole bundle began? Likely due to the addition of two crucial measures: compliance assessment and surveillance cultures with periodic feedback. To understand the relevance of the first, it should be reminded that initial observations revealed that contact precautions and hand hygiene were being poorly accomplished. Educational meetings with those responsible for environmental cleaning, the development of checklists and the direct surveillance of hand hygiene and contact precaution accomplishment started in a context of professional and institutional requirement. We opted for direct observation and checklists because of their educational effect among the several methods described to assessment processes of infection control [33].

The performance of surveillance cultures is part of most programs for the control of MDR-Ab [25–27, 29, 30, 32], supported by a moderate level of evidence [33]. The European Society of Clinical Microbiology and Infectious Diseases (ESCMID) recommend active screening cultures just for controlling the outbreaks, not for endemic situations [33]. The lack of evidence about the best anatomic site for culturing and a low sensitivity of the surveillance tools to identify the carriers have been arguments against routine screening [34]. However, a study that employed a Monte Carlo model to assess the impact of active surveillance of *A. baumannii* on patient outcomes revealed that it reduced transmissions and was cost-saving when *A. baumannii* prevalence was at least 2% and the screening test sensitivity was 55% or higher [35]. Our experience reinforces what other authors have also addressed: to eradicate or significantly reduce the incidence in an endemic situation, screening cultures are a must.

The skin is known to be a relevant reservoir of *A. baumannii,* and skin samples are frequently included in the active surveillance. Our program did not, and this could constitute a limitation of the program. There is no international consensus on the samples to be taken in the surveillance study of MDR-Ab. The guidelines of the ESCMID [33] recommend: “stool samples or swab samples from the rectum or perirectal area as well as samples from the inguinal area and manipulated sites, e.g. catheters and areas of broken skin such as wounds”, while the HICPAC/CDC guidelines recommend taking active screening cultures for MDR-gramnegative bacilli from areas of skin breakdown and draining wounds and, if a respiratory tract reservoir is suspected, from endotracheal tube aspirates or sputum [15]. In this open scenario, we chose two specimens, pharyngeal and rectal swabs, because: a) respiratory colonization and infection was the most frequently caused by *A. baumannii* among our patients; b) the sensitivity of the rectal/perianal samples can achieve a 78% (higher than the sensitivity of other localizations, as skin or wounds) [36] and the combination of pharyngeal and rectal sites can achieve a sensitivity of 96% [37]; c) several studies have related the rectal colonization by MDR-Ab with a higher risk of infection caused by this microorganism [38, 39]. Furthermore, assuming that the absence of skin screening cultures could be a limitation of the study, it did not preclude the goal of eradicating clinical colonization and infection by MDR-Ab*.*

Antimicrobial stewardship was another cornerstone of the intervention. Although some infection control programs succeeded without any specific antibiotic policy [25, 27], many others find it crucial [32, 40]. As occurs with other components of the bundles, this measure needs to be adapted to the local scenario. In our case, antibiotic pressure was likely the responsible of the XDR-Ab outbreak. A careful analysis revealed that the inappropriate duration of antimicrobial treatments and the overuse of carbapenems needed to be addressed to control either the spread of *A. baumannii* or the emergence of other MDR rods.

In our opinion, the main strengths in the design of this study are the prospective data collection and the time-series analysis, which allows the most accurate analysis in non-randomized intervention studies. This kind of analysis, as well as previous data sustaining the employed measures and no previous reports about the spontaneous disappearance of an endemic *A. baumannii*, supports the effectiveness of the presented program. On the other hand, another key factor on the tackling of this endemic situation was the multifaceted program, which reinforces our idea that a multidisciplinary and transversal team is necessary to achieve a more complete evaluation of a system of endemism, obtaining a more effective solution for a better care of patients. However, the study has also some limitations. Firstly, the design of a multifaceted program prevents us from knowing if any of the employed measures could be futile, given that all were made simultaneously, or if adding any other measure (as discussed previously for skin screening cultures) would have produced the eradication of MDR-Ab earlier. Secondly, although the present study reports data from the whole hospital, many interventions were made specifically in the ICU; thus, the extrapolation of these measures to other kind of units may require an adaptation. And finally, there are some issues with the susceptibility testing. It was not complete for all the antibiotics in all the isolates; hence, the classification as MDR/XDR/PDR relied in a number of them on the local epidemiology and the susceptibility to the main drugs. Additionally, the susceptibility to colistin was performed by E-test. In 2016, few months before the study period ended, EUCAST gave “warning” for using E-test to study the susceptibility to colistin [41], due to a risk of false susceptible results up to 32% [42]. We just started to use other susceptibility test methods after the study period. However, a possible misclassification of some isolates as “colistin-susceptible” would not modify the main results of the study.

## Conclusions

The multicomponent intervention program performed by a multidisciplinary team has been effective to control the XDR-Ab outbreak and to eradicate the endemic MDR-Ab in our hospital.

## Supplementary information

**Additional file 1.** Timeline of the measures included in the multifaceted intervention program.

## Data Availability

The data that support the findings of this study are available from the archives of the University Hospital Virgen del Rocío but restrictions apply to the availability of these data, which were used under license for the current study, and so are not publicly available. Data are however available from the authors upon reasonable request to the corresponding author (RAM) and with permission of the University Hospital Virgen del Rocío.
