# Supplementary material for: A multimodal intervention program to control a long-term Acinetobacter baumannii endemic in a tertiary care hospital
Source: Antimicrob Resist Infect Control. 2019 Dec 4;8:199. doi: 10.1186/s13756-019-0658-4 (PMC6894224; doi:10.1186/s13756-019-0658-4)
Supplement: Supplementary file 1 — Additional file 1. Timeline of the measures included in the multifaceted intervention program. [file 13756_2019_658_MOESM1_ESM.docx]

Timeline of the measures included in the multifaceted intervention program.

|  | Until Jan’11 | Jan’11-Oct’12 | Oct’12-Aug’17 |
| --- | --- | --- | --- |
|  |  |  |  |
| Weekly screening cultures (rectal and pharyngeal swab) |  |  |  |
| Exhaustive environmental cleaning policy |  |  |  |
| Contact precautions of colonized patients |  |  |  |
| Alert code to identify promptly colonized patients |  |  |  |
| Hand hygiene education program |  |  |  |
| Daily chlorhexidine baths of patients in the ICU |  |  |  |
| Antimicrobial stewardship program |  |  |  |
| Screening cultures monitoring |  |  |  |
| Environmental cleaning performance monitoring |  |  |  |
| Hand hygiene surveillance |  |  |  |
| Multidisciplinary taskforce meetings |  |  |  |
| Regular meetings with staff |  |  |  |
| Weekly feedback with the main indicators to all the staff |  |  |  |

ICU: intensive care unit.

Evolution of the consumption of diverse antimicrobials in the ICUs from the beginning of the whole intervention to the end of the study (DDD/1,000 patients-days).

|  | **2012** | **2016** |
| --- | --- | --- |
| All antimicrobials | 165.35 | 150.44 |
| Penicillins | 8.31 | 15.32 |
| Cephalosporins | 12.43 | 18.53 |
| Amoxicillin/clavulanic acid | 16.56 | 22.3 |
| Piperacillin/tazobactam | 18.99 | 20.71 |
| Carbapenems | 31.47 | 15.9 |
| Sulbactam | 1.37 | 0.03 |
| Colistin | 25.96 | 6.65 |
| Aminoglycosides | 1.43 | 2.71 |
| Quinolons | 16.37 | 17.14 |
| Tigecycline | 0.78 | 0.12 |
| Glucopeptides | 11.1 | 8.9 |
| Daptomycin | 0.61 | 0.99 |
| Linezolid | 5.51 | 4.25 |
